# Japanese honey bees (*Apis cerana japonica*) have swarmed more often over the last two decades

**DOI:** 10.1007/s00114-024-01902-y

**Published:** 2024-03-06

**Authors:** Kiyohito Morii, Yoshiko Sakamoto

**Affiliations:** National Institute for Environmental Studies, 16-2 Onogawa, Tsukuba, Ibaraki 305-8506 Japan

**Keywords:** Asian honey bee, Climate change, Global warming, Native honey bee, Reproductive behavior, Swarming cycle

## Abstract

The impacts of temperature increase are a concern for honey bees, which are major pollinators of crops and wild plants. Swarming is the reproductive behavior of honey bees that increases colony numbers. Honey bee colonies sometimes swarm multiple times, with each swarming termed a “swarming event” and a series of these events called a “swarming cycle.” The number of swarming events per swarming cycle varies widely depending on climatic conditions and subspecies, and the recent temperature increase due to global warming might be affecting the number of swarming events per swarming cycle of native honey bees. We clarified long-term changes in the number of swarming events per swarming cycle of Japanese honey bees (*Apis cerana japonica*) by collecting beekeepers’ swarming logbooks. The survey showed that between 2000 and 2022, Japanese honey bees swarmed 1 to 8 times per swarming cycle. Generalized linear model analysis indicated that year had a significant positive effect (coefficient, 0.03; 95% CI, 0.01–0.04); that is, the number of swarming events per swarming cycle showed a moderate increase over time. In addition, we found that colonies swarmed more often in a cycle when the swarming process began in early spring, especially in March. Considering the notably strong trend in Japan of warmer temperatures in March, the number of swarming events per swarming cycle may be increasing because reproduction is beginning earlier in the year. Further analyses are needed to verify the causal relationship of temperature increase on the number of swarming events per swarming cycle.

**Supplementary Information:**

The online version contains supplementary material available at 10.1007/s00114-024-01902-y.

## Introduction

Climate change is impacting global ecosystems and biodiversity (Garcia et al. 2014; Pecl et al. 2017), and understanding these effects is a defining challenge for ecology (Halsch et al. 2021). In particular, the rate of temperature increase in the twenty-first century is predicted to be the highest in the last 65 million years (Diffenbaugh and Field 2013). Climate change, including temperature increase, is affecting the behavior of various animals (e.g., Forister and Shapiro 2003; Charmantier et al. 2008; Gutiérrez and Wilson 2020). For example, in central Europe, 44 species of Lepidoptera have increased voltinism, with various ecological impacts of concern, including increased agricultural damage (Altermatt 2010). Beyond this example, temperature increase affects the life histories of many insect species (reviewed by Harvey et al. 2023). The effects of temperature increase on poikilotherms (including insects) are highly dependent on behavioral changes such as phenology, as well as shade availability (Kearney et al. 2009). Therefore, studying how rising temperatures affect animal behavior is essential for accurately assessing the impact of warming on ecosystems.

Honey bees are major pollinators of crops and wild plants (e.g., Calderone 2012; Aslan et al. 2016), contributing to the production of 39 of the leading 57 single crops (Klein et al. 2006). The impacts of climate change, including temperature increase, on important pollinator groups such as honey bees are a concern (Le Conte and Navajas 2008; Miller-Struttmann 2015; Hutchings et al. 2018; Nürnberger et al. 2019). For example, Switanek (2017) reported that hot and dry summers increase the collapse probability of *Apis mellifera* colonies in winter, and Alzate-Marin et al. (2021) demonstrated that temperature increase may alter flower-visiting behavior. In addition, analyses of long-term records of *A. mellifera* in Spain and Poland suggested that temperature increase has accelerated the timing of emergence from the hive after overwintering (Gordo and Sanz 2006; Sparks et al. 2010). However, as revealed by a meta-analysis of 293 papers by Havard et al. (2019), few studies have examined the impact of temperature increase on honey bees, especially on reproduction.

**Fig. 1:**
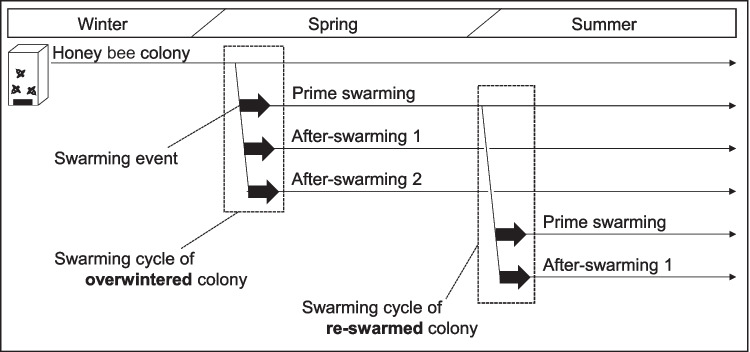
The relationship between swarming cycles of overwintered and re-swarmed colonies in this study. Thick arrows indicate each swarming event. Thin arrows indicate the presence of a colony. The dotted square represents the swarming cycle, which is a series of swarming events. This study did not distinguish whether the origin of the re-swarmed colony was prime swarming or after-swarming

Under natural conditions, members of the genus *Apis* (Apidae) only increase the number of colonies through reproductive swarming (hereafter “swarming”), in which the queen and many workers leave the original colony to establish a new nest (Grozinger et al. 2014). Honey bees sometimes swarm multiple times during a swarming cycle (i.e., a series of swarming events; see Fig. 1). In the first swarming (prime swarming), the mated queen leaves the nest with workers, and in the second and subsequent swarmings (after-swarming), a newly emerged queen leaves with workers (Winston 1980). Studies in the USA have reported that European honey bees *A. mellifera ligustica* or *A. mellifera carnica* swarm 1 to 4 times per swarming cycle (Winston 1980; Gilley and Tarpy 2005). Tropical *A. mellifera* subspecies swarm more often than temperate ones (Seeley 1985). An exhaustive study of five subspecies of *A. mellifera* in Ethiopia revealed that some subspecies swarmed an average of 10 times and up to 16 times per colony per year, whereas others swarmed an average of only 3 times (Nuru et al. 2002). It has also been reported that Africanized honey bees swarm 1 to 5 times per swarming cycle (Otis 1991). These findings indicate that the number of swarming events per swarming cycle varies with climate and subspecies, even within the same species, suggesting that the number of swarming events per swarming cycle may also be affected by temperature. A change in the number of swarming events per swarming cycle could be demonstrated by analyzing long-term records, but no such studies have been done.

The Japanese honey bee (*A. cerana japonica*), a subspecies of the Asian honey bee that is widely distributed in Asia (Engel 1999; Su et al. 2023), is a good candidate for use in large-scale research on long-term changes in the number of swarming events, unlike the European honey bee, which is generally managed to prevent natural swarming (Crane 1984). The Japanese honey bee is an endemic subspecies, distributed across Japan except for Hokkaido and Okinawa. Although Okada (1997) reported that Japanese honey bees usually swarm 1 to 2 times and sometimes up to 4 to 5 times per swarming cycle, no quantitative data on the number of swarming events per swarming cycle exist, except for reports from limited areas or a limited number of colonies (e.g., Iwasaki and Ihara 1994). Sociometric data are necessary for understanding the life history of social insects (Tschinkel 1991). However, the number of swarming events per swarming cycle is largely unknown for *A. cerana* (Hyatt 2012; Aryal 2019).

Because Japanese honey bees are more likely to abscond and produce less honey than European honey bees, they are often kept as a hobby rather than for commercial beekeeping (Yoshida 1997b, 1998). In the hobby beekeeping of Japanese honey bees, it is common to keep the bees in a frameless hive, allowing them to swarm naturally like a wild colony. For many keepers of Japanese honey bees, swarming is an opportunity to increase their colonies, so most beekeepers frequently observe the colony during the swarming season and record the dates of swarms that depart their hives. By compiling the records of the number of swarming events in each colony, we may be able to detect long-term changes in the number of swarming events per swarming cycle.

In this study, we clarified long-term changes in the number of swarming events per swarming cycle by collecting past records of the number of swarming events from keepers of Japanese honey bees. In addition, using these data we produced a frequency distribution of the number of swarming events per swarming cycle and identified the period when honey bees swarm most frequently. Considering these results, we discuss the effects of temperature increase on the number of swarming events per swarming cycle of Japanese honey bees.

## Materials and methods

### Data collection

On 23 June 2022, we emailed 239 keepers of Japanese honey bees from across Japan to ask them to share data on the number of swarming events and swarming dates for each of their colonies. We distinguish between a swarming cycle in a colony that had existed since the previous year and swarmed after overwintering (hereafter “overwintered”; Fig. 1) and one that had already swarmed earlier in the year and swarmed again within the same year (hereafter “re-swarmed”). We only used data from colonies with complete information, including the number of swarming events per swarming cycle, swarming dates, and categorization of colony status as overwintered or re-swarmed. Additionally, for all data used, we identified the beekeepers by name and the colony locations to ensure data reliability.

To distinguish between swarming cycles, it is necessary to establish a threshold for the number of days between swarming events to be considered part of the same swarming cycle. In the case of the Japanese honey bee, when a newly emerged queen is present immediately after a swarming event, the timeline until this newly emerged queen departs the hive in the subsequent prime swarm is as follows: mating flights typically occur 6 days after emergence, egg-laying begins 2–3 days after mating, larvae hatch 3 days later, queen cells are sealed 4–5 days after that, the tip of the queen’s cocoon is exposed from the queen cell as workers remove the wax cover 4 days later, and the mated queen then swarms 6–8 days after the exposing of her cocoon (Yoshida 1997a; Sasaki 1999). Therefore, in this study, swarming events with intervals of 25 days or less were considered part of the same swarming cycle.

### Statistical analysis

In all our analyses, we distinguished between overwintered and re-swarmed colonies. We calculated the average number of swarming events per swarming cycle. In addition, to investigate the temporal change in the number of swarming events per swarming cycle and in the number of days after March 1st of the prime swarm, we used a Bayesian generalized linear model (GLM) assuming a Poisson distribution. The response variable was the number of swarming events in each colony or the number of days after March 1st of the prime swarm, and the explanatory variables were the year when the swarming was observed and whether the colony was overwintered or re-swarmed. In the parameter estimation using the Markov chain Monte Carlo algorithm, 8000 steps are calculated independently four times, the first 2000 steps are removed to eliminate the influence of the initial value, and then, sampling was performed once every three steps to mitigate autocorrelation. When the $\widehat{R}$ value (Gelman et al. 2004), which is a criterion for convergence, was less than 1.1, parameters were judged to have converged. For this analysis, the rstan package (ver. 2.21.2) and the brms package (ver. 2.8.0) of R (ver. 4.0.5) were used.

We calculated the average number of swarming events for each month (March to August) in which prime swarming was recorded, to indicate the relationship between the swarming season and the number of swarming events. For these data, we performed Steel–Dwass multiple-comparison tests using the asymptotic method to calculate the probability that the number of swarming events per swarming cycle of each group was different. In addition, we calculated the intervals (days) between prime swarming and the first after-swarm events, first and second after-swarm events, second and third after-swarm events, and so on for each swarming cycle. For these data, we performed Steel–Dwass multiple-comparison tests using the Monte Carlo method. In these analyses, a *p* value ≤ 0.05 was considered significant, and the NSM3 package (ver. 1.17) of R (ver. 4.0.5) was used.

## Results

**Fig. 2:**
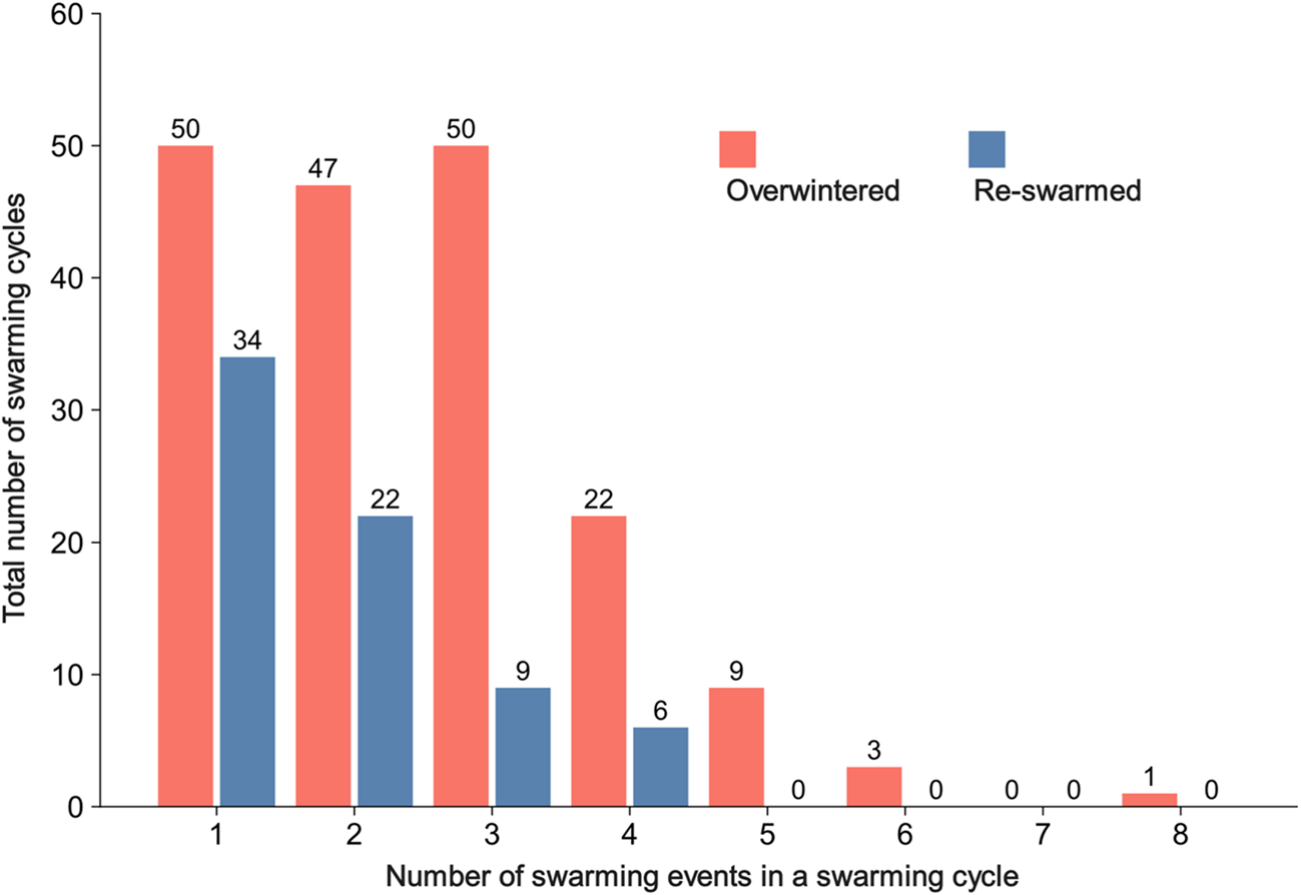
Frequency distribution of the number of swarming events in a swarming cycle. Red bars indicate overwintered colonies, and blue bars indicate re-swarmed colonies (see Fig. 1). The numbers above the bars are the total number of swarming cycles (*N* = 253)

**Fig. 3:**
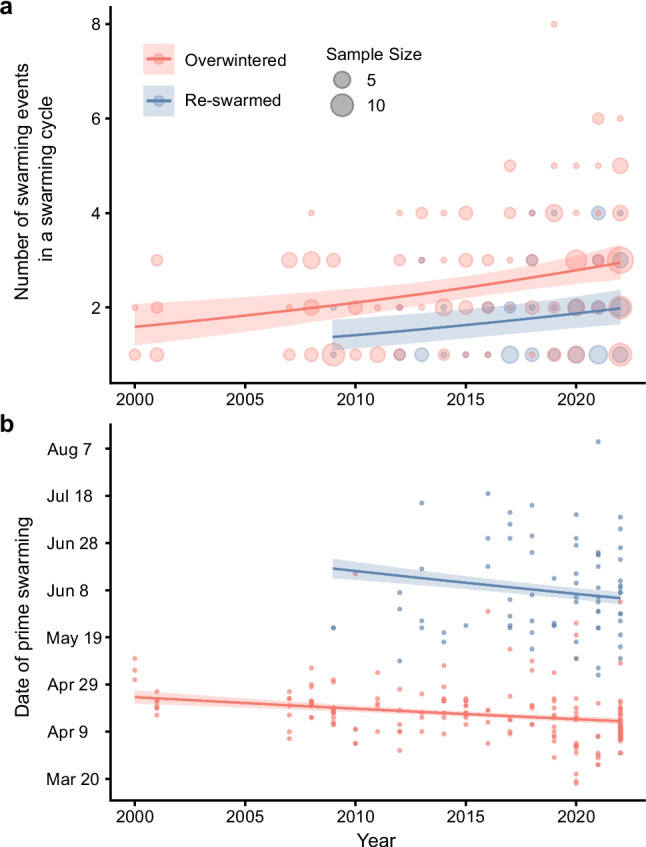
Temporal changes in the number of swarming events per swarming cycle (**a**) and the date of the prime swarm (**b**). Red and blue indicate overwintered and re-swarmed colonies, respectively (see Fig. 1). Each circle in (**a**) shows the total number of swarming events in a swarming cycle. Each data point in (**b**) shows the date of a prime swarm. The lines indicate the expected value of the number of swarming events (**a**) or the date of the prime swarm (**b**) for a given year. Shaded areas represent 95% Bayesian credible intervals for the expected value

We collected data on the number of swarming events in 253 swarming cycles between 2000 and 2022 from 20 keepers of Japanese honey bees. Japanese honey bees exhibited 1 to 8 swarming events per swarming cycle during this 23-year period (Fig. 2). In an exceptional colony that swarmed 8 times in a single swarming cycle in 2019, the second and third after-swarming events occurred on the same day, as follows: the prime swarm on March 29, the first after-swarm on April 4, the second on April 6, the third on April 6, the fourth on April 8, the fifth on April 12, the sixth on April 14, and the seventh on April 17. The overwintered and the re-swarmed colonies swarmed an average of 2.5 and 1.8 times per swarming cycle, respectively. All GLM parameters were converged ($\widehat{R}$< 1.00). The GLM analysis indicated that the number of swarming events per swarming cycle of overwintered colonies was significantly higher than that of re-swarmed colonies (coefficient, 0.40; 95% CI, 0.20 to 0.60; Fig. 3a). In addition, the analysis indicated that year had a significant positive effect (coefficient, 0.03; 95% CI, 0.01 to 0.04). That is, the number of swarming events per swarming cycle showed a moderate increase over time (by a factor of about 1.03 per year). The GLM analysis of the number of days after March 1st of the prime swarm indicated that year had a significant negative effect (coefficient, –0.01; 95% CI, –0.01 to –0.01; Fig. 3b). That is, the swarming process started earlier over time (on average, by 0.44 days per year). For the overwintered colonies in particular, the average date of the prime swarming was estimated as 24 April in 2000, and it had shifted to 13 April by 2022.

**Fig. 4:**
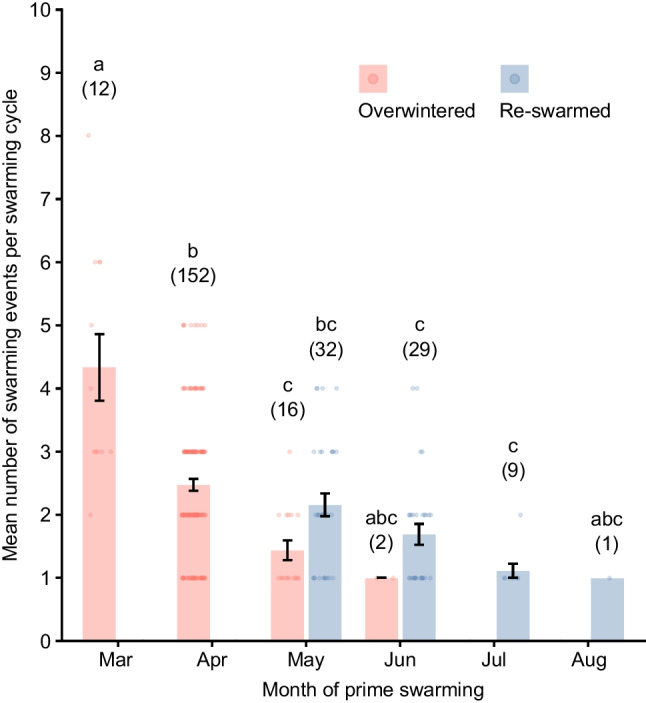
The average number of swarming events per swarming cycle in each month that prime swarming was recorded. The red and blue bars indicate overwintered and re-swarmed colonies, respectively (see Fig. 1). Each data point shows the number of swarming events in a swarming cycle. Error bars indicate standard errors. Different letters indicate a significant difference according to a Steel–Dwass multiple-comparison test (*p* < 0.05). The number in parentheses is the sample size

In both overwintered and re-swarmed colonies, the average number of swarming events was higher when the colonies swarmed earlier in the year (Fig. 4). Those overwintered colonies that swarmed in March showed a significantly higher average number of swarming events per swarming cycle than those that swarmed in the other months (Steel–Dwass multiple-comparison test, *p* < 0.05), except for months in which the sample size was 2 or less. In addition, the number of swarming events per swarming cycle in overwintered colonies that swarmed in April was significantly higher than that of May overwintered, June re-swarmed, and July re-swarmed colonies (*p* < 0.05).

**Fig. 5:**
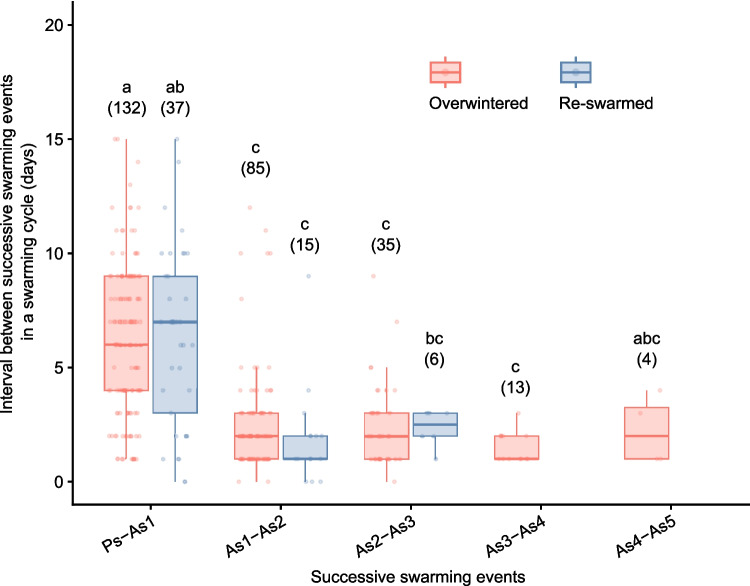
The number of days between successive swarming events within a swarming cycle. Ps and As indicate prime swarming and after-swarming, respectively. The number after As indicates the order within a swarming cycle. The red and blue boxes indicate overwintered and re-swarmed colonies, respectively (see Fig. 1). Red and blue circles show the data on which the box plot is based. The number in parentheses is the sample size. Different letters indicate a significant difference according to a Steel–Dwass multiple-comparison test (*p* < 0.05)

The median interval between the prime swarming and first after-swarming was 6 days (interquartile range, 4–9 days) in the overwintered colonies and 7 days (interquartile range, 3–9 days) in the re-swarmed colonies (Fig. 5). In contrast, the median interval between successive after-swarming events was less than 3 days. The intervals of both overwintered and re-swarmed colonies were significantly longer between the prime swarming and first after-swarming than any other combination (Steel–Dwass multiple-comparison test, *p* < 0.05), except for intervals in which the sample size was 6 or less. There was no significant difference in the intervals between successive after-swarming events.

## Discussion

This study revealed an increase in the number of swarming events per swarming cycle in Japanese honey bees between 2000 and 2022. In addition, over time, the swarming process has started earlier in the year. The colonies swarmed more often when swarming started in early spring (especially in March). Considering the notably strong trend of temperature increase in March over the last two decades in Japan, as reported by the Japan Meteorological Agency (Fig. S1), the number of swarming events per swarming cycle may have been increasing because swarming began earlier in the year. That is, the increase in winter temperatures in Japan (Fig. S2) has shortened the period during which pollen and nectar cannot be collected, allowing more overwintering bees to survive until spring, and more worker bees could result in more swarming events per swarming cycle. Asian honey bees (*A. cerana*) in tropical northern Vietnam often swarm only 1 time per swarming cycle (Chinh et al. 2005), whereas in temperate-mountainous western Pakistan, they swarm an average of 6 times and up to 10 times per swarming cycle (Ruttner et al. 1972). Japanese honey bees are a subspecies endemic to Japan and may also have the ability to swarm more often than is currently observed. If the climate continues to become warmer, the number of swarming events per swarming cycle of Japanese honey bees may continue to increase in the future.

Other studies have suggested changes in honey bee behavior due to increasing temperatures. For example, the first cleansing flight (so-called spring cleaning) of European honey bees (*A. mellifera*) in Spain and Poland now occurs earlier than it did several decades ago (Gordo and Sanz 2006; Sparks et al. 2010). Considering that European honey bees in temperate climates usually swarm in spring, it is possible that not only the number of swarming events per swarming cycle of Japanese honey bees but also that of European honey bees has increased. Likewise, changes in reproductive behavior due to temperature increase have been noted in various animal species, raising concerns about phenological mismatches with prey animals and plants (Charmantier et al. 2008; Altermatt 2010; Gutiérrez and Wilson 2020).

It is not uncommon for Japanese honey bee colonies to starve and collapse in winter because of diminished colony growth prior to winter caused by excessively repeated swarming (Matsuura 2003), indicating that an increase in the number of swarming events per swarming cycle may not necessarily result in a population increase. In studies of European honey bees, there was a positive correlation between the number of workers in a swarming event and subsequent colony growth (Lee and Winston 1987; Rangel and Seeley 2012). Theoretical studies have shown that smaller honey bee colonies are more likely to collapse (Ulgezen et al. 2021), which can result from fewer workers accompanying the queen to a new nest. European honey bees and Asian honey bees swarm more often when nectar and pollen resources are abundant (Seeley and Visscher 1985; Chinh et al. 2005), which leads to higher colony density during the swarming season when resources are abundant. When resources then become scarce, competition intensifies, and fewer colonies might survive long enough to overwinter. If we can clarify the relationship between colony size at swarming and/or the colony survival rate and number of swarming events of Japanese honey bees, we may be able to predict the effect of rising temperatures on this subspecies.

Our findings reveal several features of Japanese honey bees. First, both overwintered and re-swarmed colonies swarm more often early in the season. One hypothesis to explain this is that early swarming may improve a colony’s success when competing for nesting sites. Many European honey bee colonies in temperate areas collapse during winter (Döke et al. 2015; Seeley 2017). Similarly, in Japan, early spring is when the most vacant nest sites are available for Japanese honey bees because more than 20% of colonies fail to overwinter (or more than 50% if infected with the tracheal mite *Acarapis woodi*) between October and April (Maeda and Sakamoto 2016). Colonies that swarm early would be more likely to find high-quality nesting sites such that their fitness would be enhanced. Likewise, swarming later in the season may be less advantageous due to a relative lack of nesting sites. In addition, early swarming allows the colony enough time to stock up on resources to survive the winter (Seeley and Visscher 1985). This hypothesis could be tested by investigating changes in nesting site abundance and colony survival rate.

Second, re-swarmed colonies swarm less than overwintered colonies. One factor that may explain this is the limited amount of time available in spring and summer with good resource availability for a re-swarmed colony to recover its worker population. In addition, it may be more advantageous for re-swarmed colonies to increase the number of workers per swarming event than overwintered colonies—that is, to produce fewer after-swarms—because re-swarmed colonies are at a competitive disadvantage to overwintered colonies for nesting sites and resources. No other study has yet quantified the differences in the number of swarming events per swarming cycle between overwintered and re-swarmed colonies. Our findings highlight the need to distinguish between overwintered and re-swarmed colonies when analyzing the number of swarming events per swarming cycle of not only Japanese honey bees, but of all other honey bee populations in which re-swarming occurs within the same year.

Finally, the period between the prime swarming and first after-swarming of Japanese honey bees is longer than that of other inter-swarm intervals. The median interval between the prime swarming and first after-swarming was 6–7 days, whereas subsequent swarming events were less than 3 days apart. This might be related to the fact that the mated queen generally leaves the nest in the prime swarming (Winston et al. 1981). When more than one honey bee queen exists in a nest, they will usually kill each other until there is only one left (Gilley and Tarpy 2005). Thus, it might be adaptive for the mated queen to leave and construct a new nest before the daughter queens emerge (but for a counterexample, see Otis 1980). In Africanized honey bees, the interval between prime swarming and the first after-swarming is usually around 8–10 days (Otis 1980), slightly longer than in Japanese honey bees. This difference may arise from variations in the duration and/or process of queen rearing. Conversely, intervals between after-swarming events were similar in Africanized honey bees (Otis 1980) and Japanese honey bees, suggesting that in both species, numerous new queens are reared before prime swarming, leading to swarming at short intervals. Overall, the interval between honey bee swarming events likely follows a common rule.

This is the first report of the long-term change in the number of swarming events per swarming cycle of honey bees, providing a new perspective on the interaction between the reproductive behavior of honey bees and the environment. However, the impacts of temperature on the number of swarming events per swarming cycle of Japanese honey bees, as well as other species and subspecies of honey bees, should be verified by analyzing data from regions with various temperatures. Many interactions related to swarming, such as the relationship between the number of swarming events per swarming cycle and colony survival, as well as the proximate factors that determine the number of swarming events per swarming cycle, remain unexplained. Future studies will need to clarify the relationships between swarming events and biotic and abiotic factors and quantitatively analyze the impact of each factor.

### Supplementary Information

Below is the link to the electronic supplementary material.

Supplementary file1 (DOCX 297 KB)

Supplementary file2 (DOCX 304 KB)

## Data Availability

The datasets generated during and/or analyzed during the current study are available from the corresponding author on reasonable request.
