# Supplementary material for: Japanese honey bees (Apis cerana japonica) have swarmed more often over the last two decades
Source: Naturwissenschaften. 2024 Mar 6;111(2):14. doi: 10.1007/s00114-024-01902-y (PMC10917875; doi:10.1007/s00114-024-01902-y)
Supplement: Supplementary file 1 — Supplementary file1 (DOCX 297 KB) [file 114_2024_1902_MOESM1_ESM.docx]

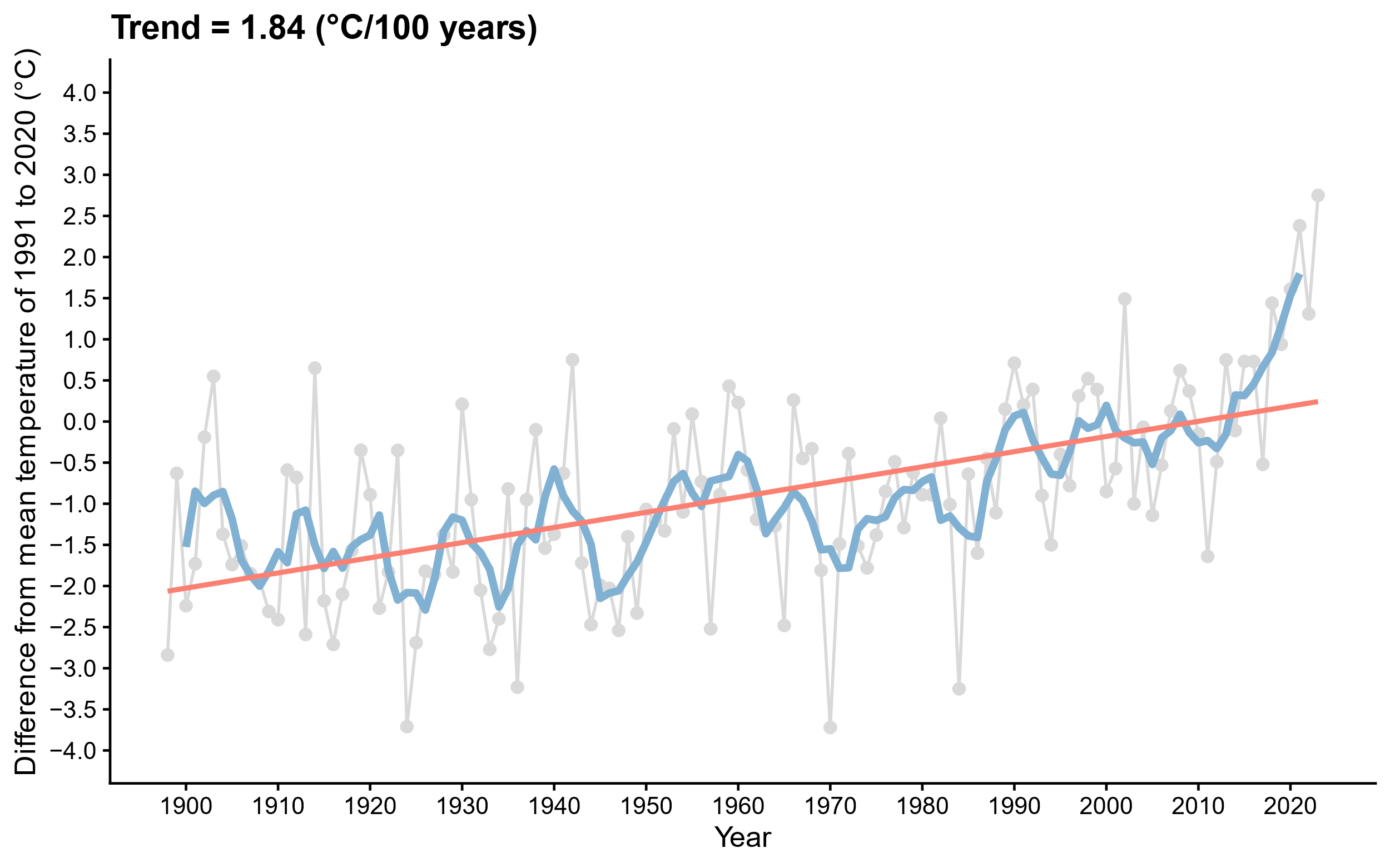


**Fig. S1** The mean temperature deviation in March in Japan from 1898 to 2023. The gray line represents the deviation from the mean temperature baseline (the average from 1991 to 2020) for each year. The blue line indicates the 5-year moving average of the deviations. The red line represents the regression line estimated using a linear model with the least squares method. This figure was created with reference to the “Monthly mean temperature in Japan” web page of the Japan Meteorological Agency (<https://www.data.jma.go.jp/cpdinfo/temp/mar_jpn.html>). The data set was downloaded from the Japan Meteorological Agency, “Monthly mean temperature deviation in Japan” (https://www.data.jma.go.jp/cpdinfo/temp/list/mon_jpn.html).
