# Supplementary material for: Japanese honey bees (Apis cerana japonica) have swarmed more often over the last two decades
Source: Naturwissenschaften. 2024 Mar 6;111(2):14. doi: 10.1007/s00114-024-01902-y (PMC10917875; doi:10.1007/s00114-024-01902-y)
Supplement: Supplementary file 2 — Supplementary file2 (DOCX 304 KB) [file 114_2024_1902_MOESM2_ESM.docx]

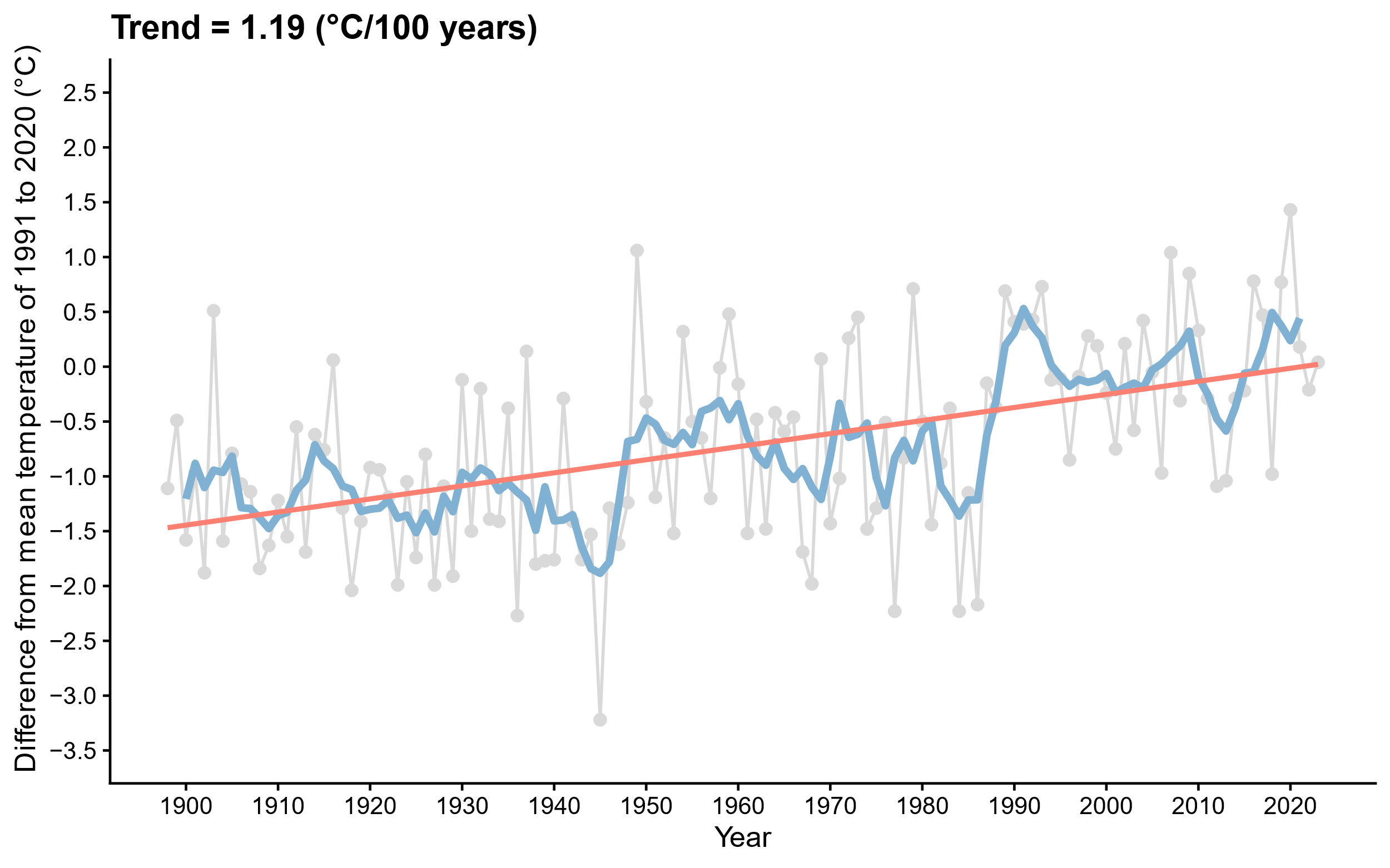


**Fig. S2** The mean temperature deviation in winter (from December to February) in Japan from 1898 to 2023. The gray line represents the deviation from the mean temperature baseline (the average from 1991 to 2020) for each year. The blue line indicates the 5-year moving average of the deviations. The red line represents the regression line estimated using a linear model with the least squares method. This figure was created by the authors with reference to the “Seasonal mean temperature in Japan” web page of the Japan Meteorological Agency (<https://www.data.jma.go.jp/cpdinfo/temp/win_jpn.html>). The data set was downloaded from the Japan Meteorological Agency, “Seasonal mean temperature deviation in Japan” (https://www.data.jma.go.jp/cpdinfo/temp/list/ssn_jpn.html).
